# Association Between Lumbar Lordosis, Thoracic Kyphosis, and Muscle Activations During Different Lower Back Exercises: An Observational Study

**DOI:** 10.3390/medicina61060986

**Published:** 2025-05-27

**Authors:** Emre Serdar Atalay, Duygu Türker, Çağlar Soylu, Tezel Yıldırım Şahan, Necmiye Ün Yıldırım

**Affiliations:** 1 Orthopedic Physiotherapy and Rehabilitation Department, Gulhane Faculty of Physiotherapy and Rehabilitation, University of Health Sciences Turkey, 34668 Ankara, Turkey; caglar.soylu@sbu.edu.tr (Ç.S.); tezelyildirim.sahan@sbu.edu.tr (T.Y.Ş.); necmiyeun.yildirim@sbu.edu.tr (N.Ü.Y.); 2 Pediatric Rehabilitation Department, Gulhane Faculty of Physiotherapy and Rehabilitation, University of Health Sciences Turkey, 34668 Ankara, Turkey; duygu.turker@sbu.edu.tr

**Keywords:** hyperlordotic and kyphotic posture, lower back extension exercises, muscle activation, observational study, young adults

## Abstract

*Background and Objectives*: Angular modifications in the physiological curvatures of the spine have been associated with spinal dysfunction and altered biomechanics, which may contribute to musculoskeletal complaints. The main objective of this experimental study was to investigate the association between lumbar lordosis and thoracic kyphosis angles and muscle activations during three different lower back exercises. *Materials and Methods*: Participants were divided into a hyperlordotic lumbar angle group—with increased thoracic kyphosis (*n* = 11)—and a normal lordotic lumbar thoracic angle group (*n* = 11). Thoracolumbar muscular activities during three different exercises were measured by surface electromyography. *Results*: Muscular activity was less in almost all exercises (except iliocostalis lumborum-pars lumborum) in the hyperlordotic lumbar angle/increased thoracic kyphosis group (*p* < 0.05). The unstable superman exercise was the exercise that produced the most muscular activity in both groups (*p* < 0.05). *Conclusions*: The study analyzed the association between lumbar lordosis, increased thoracic kyphosis, and muscle activations during specific lower back exercises. These findings highlight the relationship between spinal alignment and muscular responses during functional tasks, which may inform future biomechanical research or rehabilitation strategies.

## 1. Introduction

Alterations in the physiological curvatures of the spine have been associated with spinal dysfunction and altered biomechanics, which may contribute to musculoskeletal complaints in adolescent populations. However, the relevance of these findings to adult populations requires further investigation [1]. Angular modifications in these sagittal plane curvatures often indicate spinal disorders [2]. Using finite element modeling, Cho et al. computed that maintaining an erect sitting posture with increased lumbar lordosis during seated activities effectively reduces intradiscal pressure and cortical bone stress associated with degenerative disk diseases and spinal deformities. Although finite element modeling suggests a biomechanical benefit of increased lumbar lordosis in seated posture, the evidence directly linking lordosis to increased disk pressure remains limited, necessitating further clinical investigation [3]. Increased lumbar lordosis has been associated with elevated strain on passive spinal structures, particularly the facet joints, intervertebral disks, and posterior ligamentous complex. Such alterations may contribute to mechanical overload, altered load distribution, and potential dysfunction in spinal stability and movement patterns [4]. Additionally, viscoelastic creep in lumbar structures has been associated with pain and further dysfunction over time [5]. Spinal curvatures play a critical role in human biomechanics by optimizing energy expenditure and movement efficiency. Abnormal adaptations in thoracic and lumbar spine biomechanics, however, can lead to low back pain and dysfunction. This dysfunction may manifest as altered neuromuscular control, compensatory muscle activation patterns, and increased mechanical stress on spinal structures, which have been implicated in musculoskeletal discomfort and movement impairments [6].

A systematic review and meta-analysis found that people with low back pain display more synchronous [in-phase] horizontal pelvis and thorax rotations compared to healthy controls, highlighting significant changes in spinal biomechanics associated with lower back pain. For instance, individuals with lower back pain often exhibit altered gait mechanics [7]. Furthermore, individuals with lower back pain frequently show biomechanical alterations, such as changes in spine/trunk kinematics and muscle activity during movement tasks, which can exacerbate dysfunction over time [4,6,8].

The lumbar erector spinae muscle [LES], a superficial back muscle, spans multiple spinal segments [7]. Patients with low back pain display altered muscle activation patterns [6]. These individuals may use LES to compensate for passive ligamentous laxity, reducing the excessive forces on the lumbar spine [9]. There is evidence suggesting that individuals with chronic LBP exhibit delayed onset times of lumbar erector spinae [LES] activation compared to healthy individuals, as shown by Suehiro et al. This finding highlights the potential role of altered muscle timing in the pathophysiology of LBP [10]. For instance, a systematic review and meta-analysis reported that individuals with chronic LBP demonstrate increased LES muscle activation during forward propulsion activities [11].

Recent studies have also shown that changes in lumbar curvature may influence muscle activation patterns in individuals with chronic low back pain, with implications for both postural alignment and muscular control [12].

These findings suggest that heightened LES activation is a common characteristic among LBP patients, potentially serving as a compensatory mechanism to enhance spinal stability. Exercises targeting the LES, such as prone trunk extension, are clinically applied to strengthen these muscles [13]. A randomized controlled trial demonstrates that classification-specific treatment improved pain, disability, and fear-avoidance beliefs in individuals with lower back pain. While the study did not directly assess postural abnormalities or the prevention of spinal disorders, it highlights the potential benefits of tailored interventions for managing low back pain [14]. Strengthening the LES and thoracic extensors may reduce excessive thoracic kyphosis [15]. These exercises not only strengthen but also lengthen and stretch the muscles [16]. The hypothesis of this study is that variations in lumbar lordosis and thoracic kyphosis angles are associated with changes in muscle activations during three different low back exercises.

Recent studies have highlighted the prevalence of excessive thoracic kyphosis among athletes engaged in specific sports. For instance, a study examining adolescent female field hockey players found a significant increase in thoracic kyphosis compared to non-athletes, suggesting that the sport’s postural demands may contribute to this condition [17].

Additionally, research focusing on young athletes reported a high prevalence of postural abnormalities, including kyphosis, with 85% of the athletes exhibiting this condition [18].

Increased lumbar lordosis has been reported in dancers and gymnasts [19,20]. Previous studies indicate that muscle activation patterns, rather than lumbopelvic motion alone, play a critical role in understanding biomechanical changes. Moreover, research suggests that increased lumbar lordosis may contribute to the risk of low back pain, particularly during prolonged standing [21].

Additionally, a study found a correlation between reduced skeletal muscle mass, altered lumbar lordosis, and chronic LBP, indicating that changes in lumbar curvature may influence muscle activation patterns associated with LBP [22].

Therefore, the current research aims to investigate the relationship between spinal alignment and muscle activations during prone trunk extension exercises.

## 2. Materials and Methods

### 2.1. Study Design and Participants

This prospective study analyzed the relationship between spinal alignment and muscle activation patterns of iliocostalis lumborum (ICL) and longissimus thoracis (LT) during three low back rehabilitation exercises. It was registered in the ctv.veeva.com Protocol Registration and Results System (clinical trial number: NCT05748548). The study was approved by the University of Health Sciences Gulhane Scientific Research Ethics Committee on 22 November 2022, with approval number 2022-338/46418926) and adhered to the Declaration of Helsinki. Informed consent was obtained from all 22 participants (14 females, 8 males). It was registered in the ctv.veeva.com Protocol Registration and Results System (clinical trial number: NCT05748548, date: 3 March 2023).

Participants were split into two groups based on lumbar lordosis and thoracic posture: G1 (hyperlordotic lumbar angle- increased thoracic kyphosis, *n* = 11) and G2 (normal lordotic lumbar-thoracic angle, *n* = 11). Both groups were matched for general and medical characteristics. G1 consisted of 7 females and 4 males, while G2 consisted of 7 females and 4 males.

The age criteria for both groups was age between 18 and 24 years. Inclusion criteria for G1 were those who had lumbar lordosis angles of ≥45° and thoracic kyphosis angles ≥ 40°, while G2 it was those who had angles within normal ranges. The classification of lumbar lordosis angles of ≥45° and thoracic kyphosis angles ≥ 40° aligns with established thresholds in the literature. Normal lumbar lordosis typically ranges from 20° to 45°, with hyperlordosis often defined as an angle exceeding this range. Similarly, normal thoracic kyphosis is between 20° and 40°, with angles greater than 40° indicating hyper kyphosis. Therefore, participants in G1, with lumbar lordosis angles of ≥45° and thoracic kyphosis angles ≥ 40°, fall within these established parameters for hyperlordosis and increased kyphosis [23,24,25].

Exclusion criteria included metabolic, neuromuscular, or musculoskeletal conditions, previous spinal surgery, or participation in muscle-strengthening training within the last six months. None of the participants had experienced back or chest pain in the past year, and none reported pain during the tests.

### 2.2. Instrumentation

**Figure 1:**
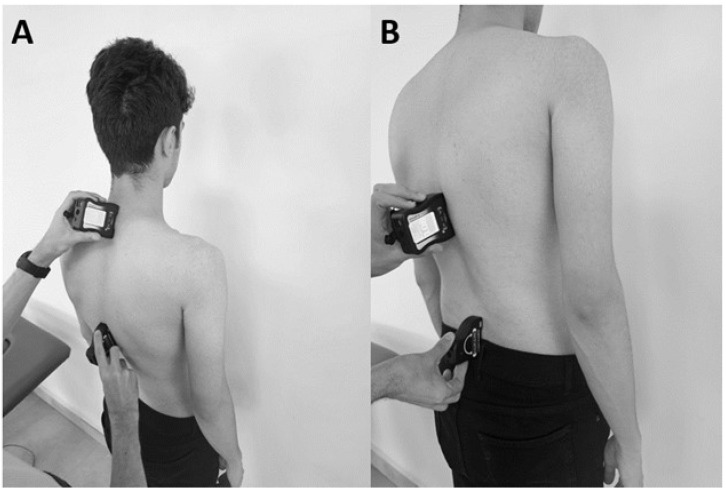
Experimental measurement of thoracic Kyphosis (**A**) and lumbar lordosis (**B**) angles.

Subjects were asked to stand with their feet shoulder-width apart and their arms relaxed at their sides, as shown in Figure 1 (Baseline^®^, Aurora, IL, USA). Measurements were made by a PhD graduate evaluator with eighteen years of experience in the physiotherapy profession. Hunter et al. demonstrated that the gravity-dependent inclinometer provides angle measurements comparable to those obtained using the modified Cobb method on radiographs, confirming its validity as a reliable and safe clinical tool for assessing thoracic kyphosis [26].

The inclinometer sensors were positioned using the spinous processes of the first thoracic vertebra (T1), the twelfth thoracic vertebra (T12), and the fifth lumbar vertebra (L5) (Figure 1). The L5 spinous process, which is the most prominent spinal process, was located above the sacrum, the T12 spinous process was located superior to the L1 point spinal process, and the T1 spinal process was located inferior to the seventh cervical vertebra. Then, using the inclinometers, the angles between T1 and T12 and between L5 and T12 were measured to evaluate thoracic kyphosis and lumbar lordosis, respectively.

#### Electromyography (EMG) Protocol

Electromyographic (EMG) data were acquired using a Noraxon Ultium EMG sensor system (Noraxon USA, Inc., Scottsdale, AZ, USA) with a sampling frequency of 4000 Hz per channel, gain of 1000, signal-to-noise ratio of 1 μV root mean square (RMS), common mode rejection ratio (CMRR) of −100 dB, and input impedance > 100 mΩ. Before electrode placement, the skin was prepared by removing hair and cleansing the area with an alcohol swab to ensure signal quality. Bipolar surface electrodes were used, placed bilaterally on the iliocostalis lumborum pars lumborum (ICL) at the L3 spinal level, the longissimus thoracis (LT) at T9, and the iliocostalis lumborum pars thoracis (ICT) at T10. The electrodes were positioned midway between the lateral-most palpable border of the erector spinae and a vertical line through the posterosuperior iliac spine, based on SENIAM guidelines, and spaced at a 2 cm interelectrode distance [27,28].

The standardization of resistance was aimed at improving comparability across subjects and minimizing variability. However, to account for individual strength differences, resistance was applied progressively, and each participant exerted their maximum voluntary effort. This approach ensured that maximal voluntary isometric contraction (MVIC) values reflected true maximal exertion rather than absolute resistance levels.

To ensure the accuracy of MVICs, investigators provided verbal encouragement during each test to maximize participant effort. Different testing maneuvers were carefully employed for each muscle group to ensure specific muscle activation. Investigators performing the MVIC tests were blinded to participant grouping to minimize bias and ensure consistency in data collection.

To minimize crosstalk from adjacent muscles, electrode placement adhered strictly to the SENIAM recommendations, and rigorous filtering was applied to the recorded data. A band-pass filter (10–500 Hz) using a first-order high-pass and fourth-order low-pass Butterworth filter was applied, along with a notch filter at 60 Hz to remove powerline noise. Additionally, potential electrocardiographic (ECG) artifacts in signals recorded from the thoracic region were filtered. The RMS values were calculated using a 100 ms moving window. Each trial’s EMG data were normalized to the mean RMS of the maximal voluntary isometric contraction (MVIC), expressed as a percentage (%MVIC). The mean %MVIC from three trials was used for analysis. The Noraxon MyoResearch XP software (version 3.16; Noraxon USA, Inc.) processed all data.

We analyzed electromyographic data from both the left and right sides for all measured muscles. For statistical evaluation, we initially tested for side differences using a repeated measures factor (side: left vs. right) in the ANOVA model.

### 2.3. Procedures

The exercise protocol consisted of three distinct phases: concentric, isometric, and eccentric. To control the speed of the concentric and eccentric phases, a 30 bpm rhythm was played with the help of a metronome, and the exercises were performed in accordance with this. The isometric phase was maintained for 10 s during each repetition (3 repetitions for each movement). Participants rested for 30 s between repetitions and took a one-minute break between exercises to mitigate fatigue. Exercises were performed in a randomized order using predefined sequences (e.g., 1-2-3, 3-2-1, 2-1-3, 2-3-1).

#### Exercise Descriptions

Prone Thoracic Extension (PTE): participants were positioned prone on a plinth, with the head aligned at the midline and arms relaxed by their sides. For the exercise, the arms were crossed over the chest, the legs remained flat on the plinth, and the trunk was extended to the maximum possible range while maintaining a neutral head position.

Superman Exercise (SU): participants were positioned prone with shoulders abducted to 180 degrees and elbows extended. Both the upper and lower extremities were lifted simultaneously off the surface to achieve a full-body extension.

Unstable Superman Exercise (USE): a Swiss ball was placed beneath the umbilicus of the participants. The feet were positioned shoulder-width apart, flat against a wall for support, and the legs were fully extended. While prone on the Swiss ball, the arms, hips, and legs were simultaneously extended to maintain a balanced position throughout the movement.

Proper form was emphasized, and real-time verbal feedback was provided to ensure consistent technique. The protocol standardized exercise intensity and reduced variability through objective timing and positional guidance.

### 2.4. Statistical Analysis

Statistical analysis was conducted using SPSS (version 26.0). *t*-test performed to determine if “general and medical characteristics” were not different. To evaluate the muscle activity differences, a three-way repeated measures ANOVA was performed. Prior to conducting the repeated measures ANOVA, assumption checks were performed. The Shapiro–Wilk test was used to assess normality, and Levene’s test was applied to evaluate the homogeneity of variances. All assumptions for parametric testing were met. The analysis included the following factors: group (two levels: intervention and control), muscle (multiple levels, representing each analyzed muscle), and exercise (multiple levels, representing different exercise types). Interaction effects between these factors (group × muscle, group × exercise, muscle × exercise, and group × muscle × exercise) were thoroughly examined. Following significant main or interaction effects, Bonferroni-corrected post hoc tests were applied to determine specific differences between conditions. These post hoc tests ensured control over Type I error while identifying significant pairwise differences

Between-group comparisons were explicitly analyzed for each muscle to assess whether group-specific differences in muscle activity existed across the exercises. This approach ensured the analysis addressed all relevant hypotheses and provided detailed insights into group, muscle, and exercise interactions. Statistical significance was set at *p* < 0.05.

## 3. Results

There were no statistically significant differences between G1 and G2 in terms of age, sex, height, or mass (*p* > 0.05). However, significant differences were observed in thoracic kyphotic angles and lumbar lordosis angles between the groups. G2 exhibited significantly lower thoracic kyphotic angles compared to G1 (*p* < 0.001, η^2^ = 0.34), while lumbar lordosis angles were significantly higher in G2 than in G1 (*p* < 0.001, η^2^ = 0.39) (Table 1). No statistically significant differences were observed between left and right muscle activation values across all exercises and groups (*p* = 0.070, η^2^ = 0.19). Therefore, for clarity and simplicity, averaged values from both sides were used in the primary analyses.

Inter-group comparisons of iliocostalis thoracis (ICT) and longissimus thoracis (LT) muscle activation revealed that G2 exhibited significantly higher activation across all exercises compared to G1 (*p* = 0.009, η^2^ = 0.28, Figure 1). This difference was most pronounced in ICT and LT, where G1 displayed significantly lower activation levels than G2 (*p* = 0.004, η^2^ = 0.31). The exercise type also had a significant effect, with the unstable superman exercise (USE) eliciting the highest activation across all muscles (*p* < 0.001, η^2^ = 0.37, Figure 1).

The interaction between muscle and exercise showed that ICT and LT activation increased progressively with exercise complexity, while ICL activation remained relatively lower across all exercises (*p* = 0.012, η^2^ = 0.25, Figure 1). Furthermore, the three-way interaction between group, muscle, and exercise confirmed that G2 exhibited consistently higher activation across all conditions, particularly in USE, whereas G1 displayed lower and more uniform activation patterns across exercises (*p* = 0.006, η^2^ = 0.29, Figure 1).

**Figure 2:**
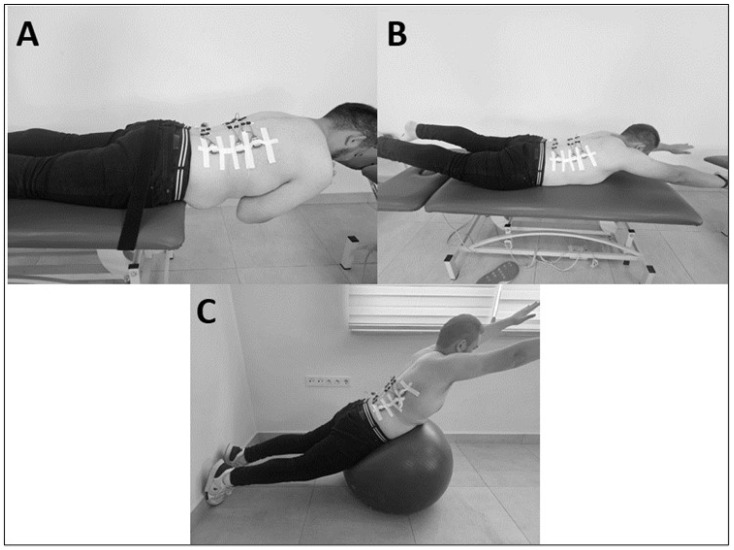
The lower back exercises. (**A**): Prone thoracic extension (PTE); (**B**): Superman exercise (SU); (**C**): Unstable superman exercise (USE).

The ICT/ICL and LT/ICL ratios were significantly different across groups and exercises (*p* = 0.008, η^2^ = 0.26, Figure 2). The group-by-exercise interaction revealed that G2 had higher ICT/ICL and LT/ICL ratios than G1 across all exercises, with the greatest difference observed in the USE condition (*p* = 0.003, η^2^ = 0.32). These findings suggest that exercise complexity plays a critical role in influencing muscle coordination and relative activation.

**Figure 3:**
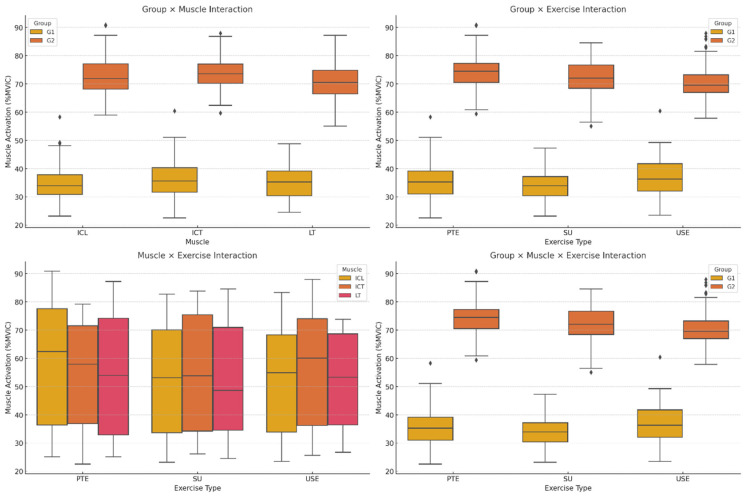
Muscle Activation Differences Across Groups and Exercises. ICT: iliocostalis thoracis; ICL: iliocostalis lumborum pars lumborum; LT: longissimus thoracis; PTE: Prone Thoracic Extension; SU: Superman Exercise; USE: Unstable Superman Exercise. Values are expressed as %MVIC: percentage of maximal voluntary isometric contraction.

The interaction between muscle ratio and exercise indicated that side differences (left vs. right) did not significantly influence ICT/ICL and LT/ICL ratios (*p* = 0.070, η^2^ = 0.19). However, the USE showed greater variability in these ratios compared to PTE and SU (*p* = 0.014, η^2^ = 0.24, Figure 2). This suggests that the instability introduced in USE may contribute to greater fluctuations in neuromuscular coordination, particularly in the LT/ICL ratio. The mean activation levels of the iliocostalis and longissimus muscles across all three exercises are presented in Figure 3.

**Figure 4:**
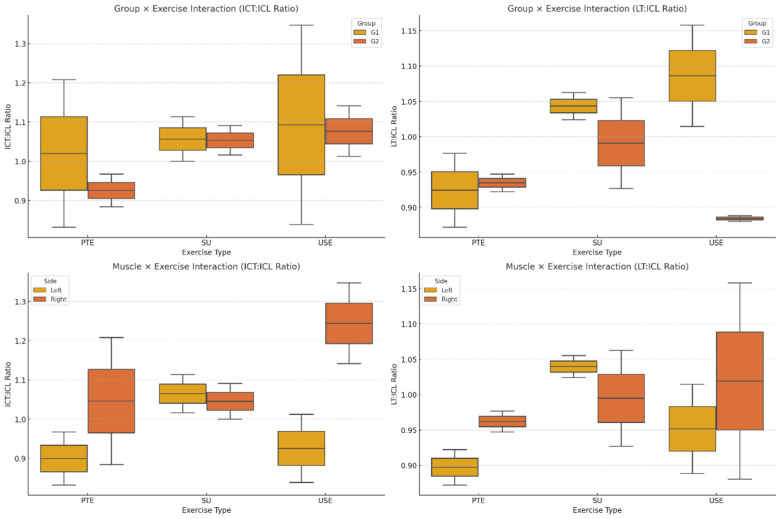
ICT/ICL and LT/ICL Ratios Across Groups and Exercises. ICT: iliocostalis thoracis; ICL: iliocostalis lumborum pars lumborum; LT: longissimus thoracis; PTE: Prone Thoracic Extension; SU: Superman Exercise; USE: Unstable Superman Exercise. Values are expressed as %MVIC: percentage of maximal voluntary isometric contraction.

Figure 4 illustrates the ratios of thoracic-to-lumbar muscle activation (ICT/ICL and LT/ICL) across the three exercise conditions in both postural groups.

## 4. Discussion

This study concluded that hyperlordosis and excessive thoracic kyphosis influence muscle activation patterns during common lower back extension exercises. Surface EMG results for the iliocostalis lumborum (IL) and longissimus thoracis (LT) muscles were analyzed during prone thoracic extension, stable superman, and unstable superman exercises.

Regarding the iliocostalis lumborum pars lumborum, the hyperlordotic/kyphotic group (G1) exhibited greater activity than the normal group (G2). This may be due to insufficient activation of thoracic extensors, requiring compensatory activation from the lumbar region. Our thoracic-to-lumbar activity ratio analysis supports this interpretation, with lower ratios observed in G1. These findings are consistent with Park et al. [27], who also reported reduced thoracic muscle activation in hyperkyphotic individuals during trunk extension. However, our study demonstrated higher effect sizes (η^2^ = 0.34–0.39), suggesting a more pronounced impact of spinal alignment on segmental muscle activation. 

Ambegaonkar et al. [19] also observed postural deviations and compensatory muscular strategies in athletes with exaggerated spinal curves, supporting our interpretation that spinal shape alters motor control strategies. Similarly, Kim et al. [28] reported that thoracic posture significantly influences erector spinae recruitment during spinal extension.

In terms of exercise type, the unstable superman exercise produced the highest muscle activation levels, consistent with prior studies, showing increased neuromuscular demand on unstable surfaces [29,30,31]. Our study expands on this by showing that individuals with altered spinal alignment may require greater stabilization effort under such conditions (η^2^ = 0.25–0.32). The interaction effect between muscle and instability (*p* = 0.006, η^2^ = 0.29) confirms the influence of complexity and segmental contribution.

Recent studies by Bervis et al. [30] and Kaur et al. [31] further emphasize that surface instability modulates trunk muscle activity by altering proprioceptive demands. However, O’Sullivan et al. [32] noted that instability does not always increase activation in seated tasks, highlighting task-specific effects of surface instability.

Although some previous reports suggest symmetrical contributions of thoracic and lumbar components [19], our data indicate that G2 participants (with normal curvatures) showed more favorable thoracic-to-lumbar ratios (η^2^ = 0.26), which may be associated with more efficient segmental synergy during extension tasks.

Current study used a convenience sample of limited size (*n* = 22). While this sample size may limit statistical power, the findings still provide preliminary insights into the associations between spinal curvature and muscle activation.

This study did not include participants with isolated kyphosis or lordosis, and future studies should investigate these subgroups separately. Additionally, although we included a validated inclinometer method for thoracic kyphosis, lumbar lordosis measurement lacked formal validation, which should be addressed in future studies.

Pelvic tilt, although not directly measured, plays a significant role in sagittal alignment and lumbar curvature. Anterior tilt is associated with increased lordosis and may influence muscle recruitment [33]. Future research should evaluate pelvic alignment as a confounding or moderating factor in postural muscle activation.

Lastly, as this was a cross-sectional analysis of acute muscle responses, long-term adaptations to exercise in individuals with spinal misalignment remain unclear. Prospective studies incorporating neuromuscular training and follow-up assessments may offer insights into rehabilitation outcomes over time.

## 5. Conclusions

The muscle response obtained from prone extension, superman, and unstable superman exercises—commonly recommended for lower back and thoracic regions—may be associated with individual lumbar and thoracic curvatures. These findings suggest that clinicians should consider individual variations in lumbar lordosis and thoracic kyphosis angles when designing rehabilitation exercise programs, as spinal alignment may significantly influence muscular responses during exercise. Given the large differences observed in comparisons of muscle activations between spinal alignment groups and across exercise types, as indicated by effect size values, posture-correcting exercises should be integrated into rehabilitation and strength training programs to enhance thoracic and lumbar muscle function. Incorporating posture-correcting exercises into programs may increase the effectiveness of these exercises for spinal muscles.

## Figures and Tables

**Table 1:** Descriptive characteristics of the participants (*n* = 22).

	G1 (*n* = 11)	G2 (*n* = 11)	
	Mean ± SD	Mean ± SD	*p* *
Age (years)	21.21 ± 1.75	21.48 ± 1.58	0.897
Height (m)	1.76 ± 0.08	1.78 ± 0.06	0.876
Mass (kg)	74.65 ± 7.04	76.07 ± 6.23	0.765
BMI (kg/m^2^)	21.04 ± 1.40	22.03 ± 1.73	0.789
Thoracic kyphotic angles (°)	44.12 ± 3.12	30.03 ± 4.84	<0.001
Lumbar lordosis angle (°)	36.41 ± 2.47	56.49 ± 3.41	<0.001

BMI: Body mass index; kg: kilogram; m: meter; (°): degree; G1: HLLA + TSP group; G2: NLLA + NTA group; SD: standard deviation; HLLA: hyperlordotic lumbar angle; NLLA: normal lordotic lumbar angle; TSP: increased thoracic kyphosis; NTA: normal thoracic angle; * independent *t*-test.

## Data Availability

The data illustrated in the present study are available on reasonable. Request from the corresponding author.
